# A Bartlett-type correction for likelihood ratio tests with application to testing equality of Gaussian graphical models

**DOI:** 10.1016/j.spl.2022.109732

**Published:** 2022-11-09

**Authors:** Erika Banzato, Monica Chiogna, Vera Djordjilović, Davide Risso

**Affiliations:** a Department of Statistical Sciences, University of Padua, via C. Battisti 241, Padua, Italy; b Department of Statistical Sciences, University of Bologna, Via Belle Arti, 41, Bologna, Italy; c Department of Economics, Ca’ Foscari University of Venice, Cannaregio 873, Venice, Italy

**Keywords:** 0000, 1111, Likelihood ratio test, Hypothesis test, Multivariate normal distribution

## Abstract

This work defines a new correction for the likelihood ratio test for a two-sample problem within the multivariate normal context. This correction applies to decomposable graphical models, where testing equality of distributions can be decomposed into lower dimensional problems.

## 1. Introduction

Testing the equality of distributions in a two sample problem can conveniently be done resorting to the likelihood ratio test (LRT) statistic, $W_{n}=-2\;\text{log}\;\Lambda_{n}$, where $\Lambda_{n}$ is the likelihood ratio. In Wilks (1938), it is shown that for samples coming from p-variate normal distributions, $W_{n}$ is asymptotically distributed as a chi-square with f=p(p+3)/2 degrees of freedom. It is well known (Muirhead, 1982) that the quality of the asymptotic approximation might be poor in finite sample problems, even at moderate sample sizes. However, convergence to the asymptotic distribution can be improved by multiplying the LRT statistic by a constant (Van der Vaart, 1998). Under the low-dimensional setting, where the number of variables p is considered fixed and n is large, the correction factor ρ proposed in Muirhead (1982) improves the convergence rate, but when the value of p is close to n or increases with it, this correction is unable to provide an improvement. In the high-dimensional setting, where p is assumed to increase with n, Jiang and Qi (2015) proposed a standardization of the LRT statistic that allows to resort to the central limit theorem and, therefore, to switch to a normal approximation. This solution, however, proves to be inaccurate for small p, given the asymmetry of the LRT statistic.

In a recent work, He et al. (2021) studied the *phase transition boundary*, d in what follows, which characterizes the approximation accuracy by establishing the necessary and sufficient condition for the chi-square approximation to hold when p increases with n. The authors showed that the chi-square approximation holds if and only if $p/n^{d}\to 0$, with  d=1/2 for the raw LRT statistic and d=2/3 for its ρ-corrected version.

In this paper, we propose a new multiplicative correction factor, $\delta_{n}$ hereafter, defined to be the ratio between the degrees of freedom of the asymptotic chi-square approximation and an approximation of the expected value of the LRT statistic, under the null hypothesis, as a function of p and n. We prove that its phase transition boundary d is equal to 1, so that the chi-square approximation holds in all situations in which p/n→0 . We show the usefulness of our proposal in the context of Gaussian graphical models (GGM). Here, the problem of testing equality of two distributions Markov with respect to a decomposable graph can be broken up into testing equality of lower dimensional Gaussian distributions. According to the structure of the graph, these lower dimensional problems can lead to very different values of the p/n ratio. Hence, it becomes crucial to rely on an approximation that guarantees a good finite sample accuracy even in extreme cases, where p is close to n.

## 2. A quick tour of the state of art

Consider two p-dimensional multivariate normal distributions, $N_{p}\left(\mu^{\left(j\right)},\Sigma^{\left(j\right)}\right)$, j=1,2, and the problem of testing their equality based on two independent random samples of size $n_{j}$. In detail, consider the hypothesis of equality of distributions

(1) $$H_{0}:\mu^{\left(1\right)}=\mu^{\left(2\right)},\Sigma^{\left(1\right)}=\Sigma^{\left(2\right)}\;\;\text{vs}.\;\;\;\;H_{a}:H_{0}\;\;\;\;\text{is}\;\text{not}\;\text{true}.$$

The LRT for testing (1), derived in Wilks (1938), can be written as

$${\text{Λ}}_{n}=\frac{{\text{Π}}_{j=1}^{2}\;\text{det}{\left({\hat{\text{Σ}}}^{\left(j\right)}\right)}^{n_{j}/2}}{\text{det}{\left(\hat{\Sigma }\right)}^{n/2}},$$

where $n=n_{1}+n_{2}$, $\hat{\Sigma }$ and ${\hat{\Sigma }}^{\left(j\right)}$*, j=1,2* are the maximum likelihood estimates of the covariance matrices under the null and alternative hypotheses, respectively, and det($\hat{\Sigma }$) denotes the determinant of $\hat{\Sigma }$. Under the null hypothesis in (1), the LRT statistic $W_{n}=-2\;\text{log}\;\Lambda_{n}$, has an asymptotic chi-square distribution, with $f=p\left(p+3\right)/2$ degrees of freedom.

In settings where p is fixed and n is allowed to grow, a first correction of the statistic $W_{n}$ was proposed by Bartlett (1937), based on a rescaling aimed at making its mean exactly equal to the mean of the asymptotic chi-square distribution, i.e., equal to f. The corrected statistic, $W_{n}^{B}$ say, takes the following form

(2) $$W_{n}^{B}=\frac{f}{E_{H_{0}}\left(W_{n}\right)}W_{n},$$

where $E_{H_{0}}\left(W_{n}\right)$ is the expected value of $W_{n}$ under the null hypothesis; see for example Van der Vaart (1998). Later, Muirhead (1982) proposed a version of Bartlett correction that leverages on an expansion of the correction factor, leading to the following correction

(3) $$\rho =1-\frac{2p^{2}+9p+11}{6\left(p+3\right)n}\left(\sum_{j=1}^{2}\frac{n}{n_{j}}-1\right).$$

The author showed that the resulting corrected statistic, $W_{n}^{\rho }$ say, where $W_{n}^{\rho }=-2\rho \;\text{log}\;\Lambda_{n}$, has a chi-square limit, with an improved approximation rate with respect to $W_{n}$. Both corrections, however, fail when p and n grow at comparable rates.

Recent studies have considered the problem when the dimension p changes with the sample size n. In these settings, Jiang and Yang (2013) and Jiang and Qi (2015) established the following result based on the central limit theorem (CLT):

(4) $$\frac{\text{log}\;\Lambda_{n}-\mu_{n}}{n\sigma_{n}}\overset{d}{\to }N\left(0,1\right),$$

where $\mu_{n}$ and $\sigma_{n}>0$ are functions of both n and p and are the asymptotic mean and standard deviation of $\text{log}\;\Lambda_{n}$, respectively. The use of the central limit theorem has the advantage of being appropriate in a high dimensional setting; however, it is less accurate when p is small, due to the asymmetric shape of the LRT distribution.

## 3. Our proposal

In this section, we propose a Bartlett-type correction of the LRT statistic, under the assumption that p changes with the sample size n. This correction replaces the denominator of (2) with a function of the approximated mean given in Eq. (4). In a two sample problem, the term $\mu_{n}$ defined by Jiang and Qi (2015) is

(5) $$\mu_{n}=\frac{1}{4}\left[-4p-\sum_{j=1}^{2}\frac{p}{n_{j}}+nr_{n}^{2}\left(2p-2n+3\right)-\sum_{j=1}^{2}n_{j}r_{{n^{\prime }}_{j}}^{2}\left(2p-2n_{j}+3\right)\right],$$

where ${n^{\prime }}_{j}=n_{j}-1$ and $r_{x}\;=\;{\left(-\log(1-p/x)\right)}^{1/2}$, for x>p, and $n=n_{1}+n_{2}$. Let $\mu_{w_{n}}=-2\mu_{n}$, we define the adjusted statistic $T_{n}$ as

(6) $$T_{n}=\delta_{n}\;W_{n},\;\;\;\;\;\delta_{n}=\frac{f}{\mu_{w_{n}}},$$

where $f=p\left(p+3\right)/2$ are the degrees of freedom of the chi-square asymptotic null distribution of $W_{n}$. We now prove that $T_{n}$ is asymptotically chi-square distributed.

**Theorem 1.** *Let $\text{p}={\left(p_{n}\right)}_{n\in N}$ be a sequence of integers $1\leq p_{n}<n_{j}-1$. Under $H_{0}$, for $T_{n}$ defined as in* (6)*, $\text{mi}{\text{n}}_{j=1,2}\;n_{j}\to \infty$ and p/n→0, we have that*

$$\underset{-\infty <x<\infty }{\text{sup}}\left|P\left(T_{n}<x\right)-P\left(\chi_{f_{n}}^{2}<x\right)\right|\to 0$$

and the phase transition boundary of $T_{n}$ is d=1.

**Proof.** See Appendix A. □

In Theorem 1, the condition $n_{j}>p+1$ is assumed to ensure the existence of the LRT. Moreover, the condition p/n→0 defines the phase transition of the adjusted statistic, as introduced in He et al. (2021), which represents the boundary in which the chi-square approximation starts to fail as p increases and characterizes the approximation accuracy. This boundary is an improvement over $W_{n}$ and $W_{n}^{\rho }$, whose approximations hold for ${p/n}^{d}\to 0$, with d=1/2 and d=2/3, respectively.

## 4. Simulation study

In this section we present a simulation study to compare the performances of the LRT statistics based on four different approximations: the classic chi-square approximation, the ρ-adjusted approach of Muirhead (1982), the CLT approach of Jiang and Qi (2015) and our proposed δ-adjusted approach.

**Fig. 1.**
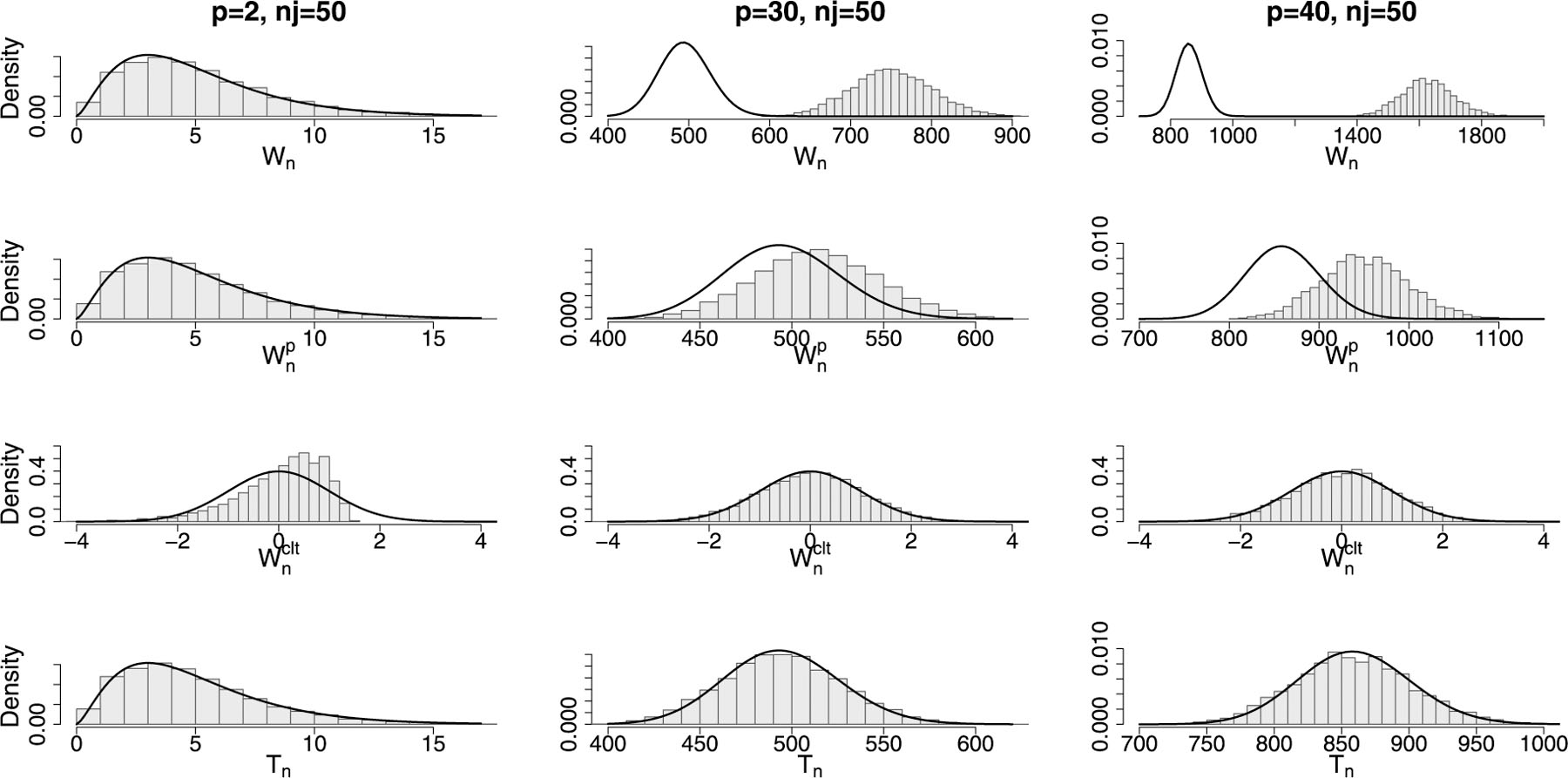
Simulation results with $n_{1}=n_{2}=50$ and p=2,  30,  40. From the top to the bottom row: empirical distribution of $W_{n}$, $W_{n}^{\rho }$, $W_{n}^{clt}$, and $T_{n}$. The solid line in the first, second, and fourth rows shows the nominal $\chi^{2}$ distribution, with 5, 495 and 860 degrees of freedom (from left to right) respectively. The solid line in the third row, corresponding to the $W_{n}^{clt}$ statistic, shows the standard normal distribution.

We study how the correction acts considering a fixed sample size and letting the dimension p change. Data are drawn from a multivariate normal distribution, with fixed covariance matrix and mean vector and we set $n_{1}=n_{2}=50$ and p=2, 30, 40. For each scenario, five thousand simulations are run. Results are shown in Fig. 1. For each value of p we plot the histograms of the empirical distribution of the four statistics, namely $W_{n}$, $W_{n}^{\rho }$, $T_{n}$ and $W_{n}^{clt}$, and compare them with the chi-square distribution with $p\left(p+3\right)/2$ degrees of freedom in the first three cases and a standard normal in the last case. The top row of Fig. 1 shows how the statistic $W_{n}$ departs from the theoretical $\chi^{2}$ distribution as p grows. This is expected and motivates the need of an adjustment when dealing with testing problems in which the dimension grows with n. In fact, if 50 observations might be enough for testing a problem of dimension 2, this is not the case for other values of p, especially when p and n have comparable values. The second row shows the results for the statistic corrected with ρ. Note that, also in this case, the approximation to the $\chi^{2}$ fails as p approaches the group sample size, $n_{j}$. With respect to the previous case, however, the departure from the chi-square distribution occurs for higher values of p. The third row highlights the problem of applying the CLT when p is small. For example, when p=2 the approximation to the normal distribution fails, while it improves as p increases. This approach works well also for values of p very close to $n_{j}$. The bottom row shows the accuracy of the approximation of the proposed adjusted statistic $T_{n}$. Note that this correction leads to a good approximation regardless of the dimension of the testing problem, as long as p/n→0, and could be used as a unique tool for correcting $W_{n}$ at different values of p and n.

**Fig. 2.**
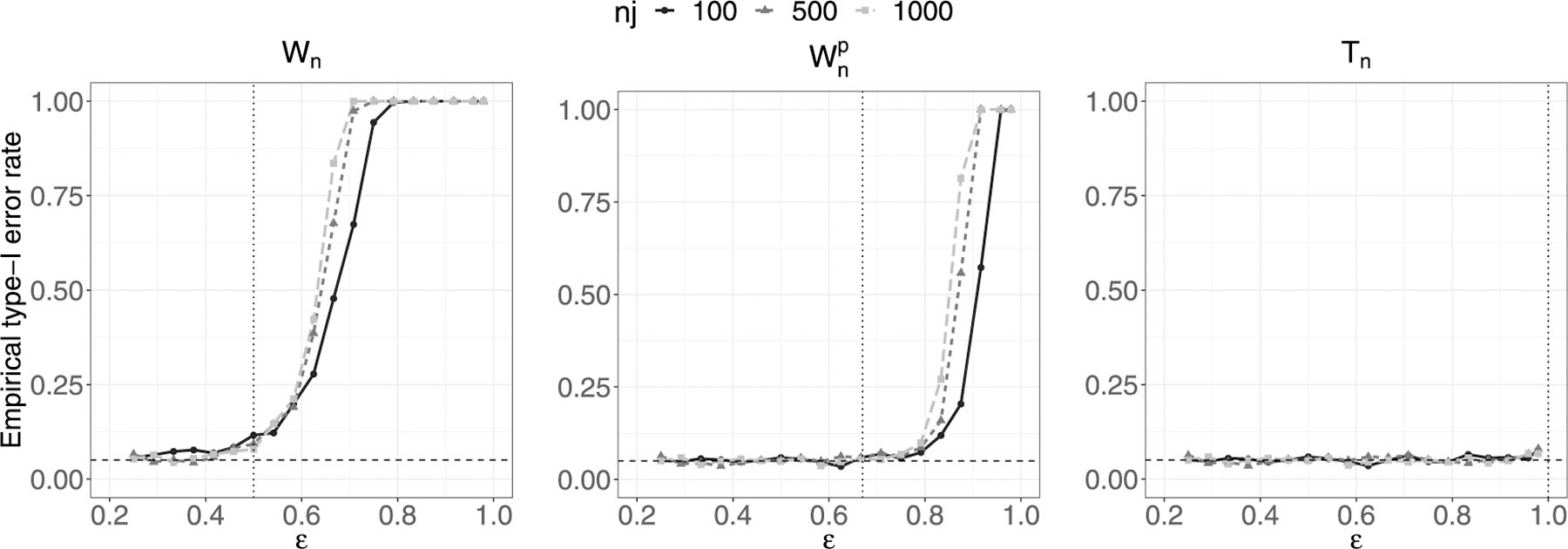
Chi-square approximation of $W_{n}$, $W_{n}^{\rho }$ and $T_{n}$. Empirical type-I error rate for $n_{j}\in \left\{100,\;500,\;1000\right\}$, j=1,2 over 1000 simulations. The vertical dotted lines represents the phase transition boundaries for the three statistics: 1*/*2, 2*/*3 and 1, respectively. The horizontal dashed line represents the nominal significance level, 0.05.

Finally, we run some simulations to examine the phase transition boundary in Theorem 1, under the null hypothesis. We consider $p=\left\lfloor n_{1}^{\varepsilon }\right\rfloor$, $n_{1}=n_{2}$, $n=\sum_{j=1}^{2}n_{j}$ and $n_{j}\in \left\{100,500,1000\right\}$ and finally $\varepsilon \in \left\{6/24,\ldots ,23/24,23.5/24\right\}$. $\left\lfloor \cdot \right\rfloor$ denotes the rounding to the nearest integer function. We plot the empirical type-I error rate (over 1000 simulations) versus ε, for each chi-square approximation: $W_{n}$, $W_{n}^{\rho }$ and $T_{n}$. Results are plotted in Fig. 2. The first two panels confirm the results in He et al. (2021), while the one on the right hand side shows how the phase transition boundary of the adjusted statistic $T_{n}$ is close to 1. The particular case with ε exactly equal to one is excluded, to ensure the identifiability of the covariance matrix.

## 5. Testing equality of distributions in Gaussian graphical models

In the remaining sections of the paper, we assume the reader is familiar with the basic theory of (decomposable) undirected graphical models, as presented for instance in Lauritzen (1996); see also Whittaker (1990). We adopt a standard terminology and a rather intuitive notation: we let $G=\left(V,E\right)$ denote an undirected graph, with V a finite set of nodes and $E=\left\{\left(v,t\right):v\neq t;\;\;v,t\in V\right\}$ a finite set of edges between vertices. We denote its cliques, separators and residuals by C, S and R, respectively.

Our proposal finds a natural application in the context of decomposable graphical models. One prominent advantage of decomposable graphs is that their cliques can be arranged so as to satisfy the running intersection property (RIP), and the joint probability distribution of the associated random vectors factorizes accordingly. In detail, if a graph $G=\left(V,E\right)$ decomposes into k, say, cliques, let $C_{i}$, i=1,…,k, be a sequence of cliques satisfying the RIP and $S_{i}=C_{i}\cap C_{i-1}$ and $R_{i}=C_{i}\backslash C_{i-1}$, i=2,…,k the set of corresponding separators and residuals, respectively. Then, the probability distribution of the random vector $X_{V}$ factorizes as $f\left(X_{V}\right)=f\left(X_{C_{1}}\right)f\left(X_{R_{2}}\left|X_{S_{2}}\right.\right)\ldots f\left(X_{R_{k}}\left|X_{S_{k}}\right.\right)$. See Lauritzen (1996) for an exhaustive explanation. Such factorization renders tractable inference in the setting of large-scale graphical models, where the dimension p of the problem is higher that the available sample size n. Even when p<n, using the information on the graphical structure allows us both to improve the power of detecting a difference between the two distributions under study (the size of the model is reduced by constraints on the covariance matrix), and to localize that difference, thanks to the modular nature of graphical models (Djordjilović and Chiogna, 2022). This potential has fed the increasing prominence of graph–theoretic representations of probability distributions in fields such as statistical and quantum physics, bioinformatics, signal processing, econometrics and information theory. In our problem setting, this factorization assumes a crucial role as it allows to decompose the global problem of testing equality of distribution in two samples into a sequence of local tests of equality of distributions defined on a smaller set of variables, as follows

(7) $$H=\overset{k}{\underset{i=1}{\cap }}H_{i},\;\;\;\;\;\;H_{i}:X_{R_{i}}^{\left(1\right)}\left|X_{S_{i}}^{\left(1\right)}\overset{d}{=}X_{R_{i}}^{\left(2\right)}\right|X_{S_{i}}^{\left(2\right)},\;\;\;\;\;i=1,\ldots ,k,$$

with $S_{1}=\emptyset$ and $R_{1}=C_{1}$. Hence, to test the global hypothesis H, one can test the k local hypotheses $\left\{H_{i},i=1,\ldots ,k\right\}$ of equality of the conditional distributions of $X_{R_{i}}\left|X_{S_{i}}\right.$. In the case of strong meta Markov models (Lauritzen, 1996; Edwards, 2000), as is the Gaussian case, Djordjilović and Chiogna (2022) showed that the local hypotheses $H_{i}$*, i=1,…,k*, are independent and that the LRT statistic for testing H also decomposes into k LRT statistics, one for testing each local hypothesis. Specifically, the LRT, $W_{n}$, factorizes as

(8) $$W_{n}=\sum_{i=1}^{k}\left[W_{n}^{C_{i}}-W_{n}^{S_{i}}\right]=W_{n}^{C_{1}}+\sum_{i=2}^{k}W_{n}^{C_{i}\left|S_{i}\right.},$$

where $W_{n}^{A}$*, A⊆V*, represents the LRT for the hypothesis of equality of distributions for $X_{A}$, namely $H_{\left(A\right)}:\mu_{A}^{\left(1\right)}=\mu_{A}^{\left(2\right)}$, $\Sigma_{A}^{\left(1\right)}=\Sigma_{A}^{\left(2\right)}$, while $\;W_{n}^{A\left|B\right.}$ is the LRT for the hypothesis of equality of distributions for $X_{A}\left|X_{B}\right.$*, B⊆V\A*, namely $H_{\left(A\left|B\right.\right)}:\mu_{\left(\;A\left|B\right.\right)}^{\left(1\right)}=\mu_{\left(A\left|B\right.\right)}^{\left(2\right)}$, where $\mu_{\left(A\left|B\right.\right)}=\mu_{A}-\Sigma_{AB}\Sigma_{B}^{-1}\mu_{B}$ and $\Sigma_{\left(A\left|B\right.\right)}=\Sigma_{A}-\Sigma_{AB}\Sigma_{B}^{-1}\Sigma_{BA}$. As proved in Theorem 1 of Djordjilović and Chiogna (2022), the k statistics $W_{n}^{C_{1}}$ and $W_{n}^{C_{i}\left|S_{i}\right.}$*, i=2,…,k*, in the right-hand side of (8) are all asymptotically independent and chi-square distributed, with $f_{C_{1}}$ and $f_{C_{i}}-f_{S_{i}}$*, i=2,…,k*, degrees of freedom, respectively, being $f_{C_{i}}$ and $f_{S_{i}}$ the degrees of freedom associated to the marginal test on the cliques and the separators, respectively. It is worth noting that, since $W_{n}^{A\left|B\right.}=W_{n}^{A}-W_{n}^{B}$, the only quantities needed to compute $W_{n}$ are the observed values of the LRT on the marginal distributions defined over cliques and separators. It is easy to see that

(9) $$W_{n}^{A}=\sum_{j=1}^{2}n_{j}\;\text{log}\frac{\text{det}\left({\hat{\Sigma }}_{A}\right)}{\text{det}\left({\hat{\Sigma }}_{A}^{\left(j\right)}\right)}$$

for $A\in \left\{C_{1},\ldots ,C_{k},\;\;S_{1},\ldots ,S_{k}\right\}$. Here, ${\hat{\Sigma }}_{A}$ is the maximum likelihood estimate of $\Sigma_{A}$, the block submatrix corresponding to the nodes in A in the null covariance matrix $\Sigma =\Sigma^{\left(1\right)}=\Sigma^{\left(2\right)}$; and ${\hat{\Sigma }}_{A}^{\left(j\right)}$ are the maximum likelihood estimates of ${\hat{\Sigma }}_{A}^{\left(j\right)}$, the block submatrices corresponding to the nodes in A of $\Sigma^{\left(j\right)}$*, j=1,2*. Moreover, each $W_{n}^{A}$ has a chi-square limit with $f_{A}=p_{A}\left(p_{A}+3\right)/2$ degrees of freedom, where $p_{A}$ is the cardinality of the set A. One remarkable side effect of the decomposition is that the dimension of each local problem is determined by the cardinality of the set of variables on which it is defined, so that, for a fixed sample size n, dimensionality regimes of local problems vary as a function of their cardinality. Local problems for which p≪n might coexist with problems for which p≈n.

Our proposal naturally steps in this context, providing a convenient solution able to accommodate such variety of situations. The extension of our correction to the test statistics of the kind $W_{n}^{C\left|S\right.}$ does not represent an obstacle, resulting indeed to be straightforward. In fact, being $E\left(W_{n}^{C\left|S\right.}\right)=E\left(W_{n}^{C}\right)-E\left(W_{n}^{S}\right)$, it results $\mu_{n}^{C\left|S\right.}=\mu_{n}^{C}-\mu_{n}^{S}$. The corrected statistics for the tests relative to the decomposition (7) simply become

(10) $$T_{n}^{C_{1}}=\delta_{n}^{C_{1}}W_{n}^{C_{1}},\;\;\;\;\;\delta_{n}^{C_{1}}=\frac{f_{C_{1}}}{\mu_{n}^{C_{1}}}$$

(11) $$T_{n}^{C_{i}\left|S_{i}\right.}=\delta_{n}^{C_{i}\left|S_{i}\right.}W_{n}^{C_{i}\left|S_{i}\right.},\;\;\;\;\;\delta_{n}^{C_{i}\left|S_{i}\right.}=\frac{f_{C_{i}\left|S_{i}\right.}}{\mu_{n}^{C_{i}\left|S_{i}\right.}},\;\;\;\;\;i=2,\ldots ,k.$$

## 6. Simulation in the graphical setting

In this section, we present a simulation study aimed at showing the performances of our corrected LRTs versus ordinary LRTs when working with Gaussian graphical models. For a real data application, see the Supplementary Material. We consider a p-variate Gaussian graphical model Markov with respect to a graph with p=14 nodes and k=4 cliques (see Supplementary Material for a representation of the graph). We consider a RIP-respecting sequence $C_{1}$*, $C_{2}$, $C_{3}$, $C_{4}$* of cliques, with cardinalities $\left|C_{1}\right|=8$*, $\left|C_{2}\right|=5$, $\left|C_{3}\right|=3$, $\left|C_{4}\right|=2$*, giving rise to the following cardinalities for the corresponding sequence of separators: $\left|S_{2}\right|=2$*, $\left|S_{3}\right|=1$, $\left|S_{4}\right|=1$*. We generate data assuming that differences between the two conditions are attributable to nodes 1 and 2, located in $C_{1}$. In particular, in one condition the means of the two elected nodes is set to be 1.5 times greater than the means of the same nodes in the other condition, while the variances are decreased by 50%. It follows that the null hypothesis of equality of distribution for $X_{C_{1}}$ is false, since $C_{1}$ includes the two altered nodes. All remaining null hypotheses of equality of distribution for $X_{R_{i}}\left|X_{S_{i}}\right.$*,  i=2, 3, 4*, are true, thanks to the Markov properties of the graph. We run 10,000 simulations assuming $n_{1}=n_{2}\in \left\{10,\;50,\;100,\;250\right\}$. For each sample, we compute the following statistics: $W_{n}^{C_{1}}$, $W_{n}^{C_{i}\left|S_{i}\right.}$, $T_{n}^{C_{1}}$, $T_{n}^{C_{i}\left|S_{i}\right.}$*,  i=2, 3, 4*. The nominal Type I error rate is set to be α=0.05.

Results are reported in Table 1 (see also the Supplementary Material for a simulation under the global null). Row 1 of Table 1 shows the empirical power of the test, while rows 2–4 show the empirical Type I error rates. For what concerns $W_{n}$, note that for small sample sizes, the empirical Type I error rate is significantly higher than the nominal one, due to a large number of false rejections. This happens for all the local problems, but, for a fixed sample size, the number of false rejections largely depends on the dimension of the problem. As expected, this behavior decreases as the sample size increases, and asymptotically, the distribution of $W_{n}$ can be approximated with a chi-square. On the other hand, the adjusted statistic $T_{n}$ reaches the nominal size of the test for each considered sample size, regardless of the dimension of the local problems. The power of the test based on the adjusted statistic $T_{n}$ on the clique $C_{1}$ increases with the sample size. The high power observed for $W_{n}$ should not be misleading, as it highly depends on the false rejections due to the approximation issues already highlighted in Section 4. The adjusted statistic meets the expectations, being able to identify the altered clique, while controlling the Type I error of the remaining local tests.

## 7. Conclusions

In this paper, we proposed an adjusted LRT, which leads to valid inference at different dimensionality regimes. Our proposal overcomes some weaknesses of alternative corrections reported in the literature, that occur at small sample sizes and, in particular, when the dimension p is close to n. We showed that the phase transition boundary of the LRT statistic corrected following our proposal is d=1, indicating that the only condition needed to work is p/n→0. Simulations confirmed that the adjusted test statistic is well approximated by a chi-square distribution both for small and large values of p.

In the context of decomposable Gaussian graphical models, where the problem of testing equality of two networks breaks down into a sequence of problems defined on smaller sets of variables, our correction can help tackling the possibly high heterogeneity resulting from the decomposition in terms of dimensionality regimes. Our simulation study showed that the size of the test was reached for different configurations of p and n and, in the presence of a difference in two conditions, the adjusted statistic is able to detect it, still controlling the Type I error in the other cliques.

## Supplementary Material

Supplementary file

## Figures and Tables

**Table 1:** Power and Type I error computed for each term of the decomposition. Proportion of rejected tests out of 10 thousand simulations, for different sample sizes, with significance level α=0.05.

$n_{j}$	$W_{n}$				$T_{n}$			
	10	50	100	250	10	50	100	250
$C_{1}$	0.985	0.730	0.970	1.000	0.066	0.535	0.946	1.000
$C_{2}\left|S_{2}\right.$	0.445	0.082	0.065	0.056	0.048	0.051	0.050	0.049
$C_{3}\left|S_{3}\right.$	0.167	0.061	0.056	0.051	0.049	0.044	0.048	0.049
$C_{4}\left|S_{4}\right.$	0.109	0.060	0.051	0.057	0.047	0.052	0.048	0.055

## Data Availability

The data are publicly available in the R package ALL.
