# Supplementary material for: A Bartlett-type correction for likelihood ratio tests with application to testing equality of Gaussian graphical models
Source: Stat Probab Lett. Author manuscript; Available in PMC 2024 Apr 5. (PMC10997343; doi:10.1016/j.spl.2022.109732)
Supplement: Supplementary file [file NIHMS1913143-supplement-Supplementary_file.pdf]

Supplementary material

A Bartlett-type correction for likelihood ratio tests with application to testing equality of Gaussian graphical models

Erika Banzato[a], Monica Chiogna[b], Vera Djordjilović[c], Davide Risso[a]

[a]Department of Statistical Sciences, University of Padua, via C. Battisti 241, Padua, Italy
[b]Department of Statistical Sciences, University of Bologna, Via Belle Arti, 41, Bologna, Italy
[c]Department of Economics, Ca' Foscari University of Venice, Cannaregio 873, Venice, Italy

---

Appendix A. Proof of Theorem 1

First of all, let $T_n = \delta_n W_n$ as define in (6), with $\delta_n = f/\mu_{w_n}$ anf $f = p(p+3)/2$. Define now the two main quantities μ_{w_n} and σ_{w_n}, respectively mean and variance of W_n, from the quantities defined in Jiang and Qi (2015), for the specific case of comparison of two populations. Let

$$\mu_{w_n} = \frac{1}{2}\left[4p + \sum_{j=1}^{2}\frac{p}{n_j} + n(2p-2n+3)\log\left(1-\frac{p}{n}\right) - \sum_{j=1}^{2} n_j(2p-2n_j+3)\log\left(-\frac{p}{n_j-1}\right)\right]$$

$$\sigma^2_{w_n} = 2n^2\left[-\sum_{j=1}^{2}\frac{n_j^2}{n^2}\log\left(1-\frac{p}{n_j-1}\right) + \log\left(1-\frac{p}{n}\right)\right]$$

where $n = n_1 + n_2$. Hence, $E(T_n) = f$ and $Var(T_n) = \sigma^2_{T_n} = \frac{f^2}{\mu_{w_n}}\sigma^2_{w_n}$.

We prove Theorem (1) under two assumptions:

1. $p_n = p$ fixed.
2. $\lim_{n\to\infty} p_n = \infty$

Assumption 1. $f_n = f$ is a fixed integer, such that $f = p(p+3)/2$. It suffices to show that T_n converges in distribution to a χ^2_f. First of all, we show that $f/\mu_{w_n} \to 1$. We use $\log(1-x) = -x - x^2/2 - x^3/3 - x^4/4 + O(x^5)$ and write

$$\begin{aligned} n(2p-2n+3)\log\left(1-\frac{p}{n}\right) &= n(2p-2n+3)\left(-\frac{p}{n} - \frac{p^2}{2n^2} - \frac{p^3}{3n^3} - \frac{p^4}{4n^4} + O\left(\frac{p^5}{n^5}\right)\right) \\ &= 2pn - 3p - p^2 - \frac{p^3}{3n} - \frac{3p^2}{2n} - \frac{p^4}{6n^2} - \frac{3p^4}{4n^3} + O\left(\frac{p^5}{n^3}\right) \end{aligned} \tag{A.1}$$

Similarly, by using Taylor's expansion and $n_j = \Theta(n)$ and using $1/(n_j-1) = 1/n_j + 1/n_j^2 + o(n_i^{-3})$ and $1/(n_j-1)^a = 1/n_j^a + o(n_i^{-3})$ for $a \geq 2$ we have

$$\begin{aligned} n_j(2p-2n_j+3)\log\left(1-\frac{p}{n_j-1}\right) &= \\ &= n_j(2p-2n_j+3)\left(-\frac{p}{n_j-1} - \frac{p^2}{2(n_j-1)^2} - \frac{p^3}{3(n_j-1)^3} + O\left(\frac{p^4}{n^4}\right)\right) \\ &= n_j(2p-2n_j+3)\left(-\frac{p}{n_j} - \frac{p}{n_j^2} - \frac{p^2}{2n_j^2} - \frac{p^3}{3n_j^3} + O\left(\frac{p^4}{n^3}\right)\right) \\ &= p^2 + p - 2pn_j + \frac{3p}{n_j} + \frac{7p^2}{2n_j} + \frac{p^3}{n_j} + \frac{p^3}{3n_j^2} + O\left(\frac{p^4}{n}\right) \end{aligned} \tag{A.2}$$

Hence, as $n \to \infty$, using (A.1) and (A.2) we have

$$\begin{aligned} \mu_{w_n} &= \frac{1}{2}\left[4p - 3p - p^2 + 2p^2 + 2p + o(n^{-1})\right] \\ &= p(p+3)/2 + o(1) = f + o(1). \end{aligned} \tag{A.3}$$

Then, $T_n = W_n(1+o(1))$ and using Slutsky theorem we have that since $W_n \xrightarrow{d} \chi^2_f$, also $T_n \xrightarrow{d} \chi^2_f$.

Assumption 2. If $p_n \to \infty$, as a consequence, also $f_n = p_n(p_n+3)/2 \to \infty$ and we can write

$$\lim_{n\to\infty}\sup_x |P(T_n < x) - P(\chi^2_{f_n} < x)| = \lim_{n\to\infty}\sup_x \left| P\left(\frac{T_n - f_n}{\sigma_{T_n}} < x\right) - P\left(\frac{\chi^2_{f_n} - f_n}{\sigma_{T_n}} < x\right)\right|$$
$$= \lim_{n\to\infty}\sup_x \left| P\left(\frac{T_n - f_n}{\sigma_{T_n}} < x\right) - \phi(x) + \phi(x) - P\left(\frac{\chi^2_{f_n} - f_n}{\sigma_{T_n}} < x\right)\right| \tag{A.4}$$

$(T_n - f_n)/\sigma_{T_n}$ converges in distribution to a $N(0,1)$ because $(T_n - f_n)/\sigma_{T_n} = (-2\log\Lambda - \mu_n)/\sigma_n \to N(0,1)$ as shown in Jiang and Qi (2015). Moreover, applying Berry-Esseen theorem to $\chi^2_{f_n}$ variable we obtain

$$\lim_{n\to\infty}\sup_x \left| P\left(\frac{\chi^2_{f_n} - f_n}{\sqrt{2f_n}} < x\right) - \phi(x)\right| \to 0 \tag{A.5}$$

Hence, to show (A.4) it is enough to prove that $\sigma^2_{T_n}/(2f_n) \to 1$ as $n \to \infty$. Using (A.1) and (A.2) μ_{w_n} can be written as

$$\begin{aligned}\mu_{w_n} =& \frac{1}{2}\left\{4p + \sum_{j=1}^{2}\frac{p}{n_j} + 2pn - 3p - p^2 - \frac{p^3}{3n} - \frac{3p^2}{2n} - \frac{p^4}{6n^2} - \frac{3p^4}{4n^3} + O\left(\frac{p^5}{n^3}\right)\right. \\ & 2p^2 + 2p - 2pn + \sum_{j=1}^{2}\left[3\frac{p}{n_j} + 7\frac{p^2}{2n_j} + \frac{p^3}{n_j} + \frac{p^3}{3n_j^2} + O\left(\frac{p^4}{n_j}\right)\right] \\ =& \frac{1}{2}\left[3p + p^2 + O\left(\frac{p}{n}\right) + O\left(\frac{p^2}{n}\right) + O\left(\frac{p^3}{n}\right) + O\left(\frac{p^3}{n^2}\right)\right] \\ =& \frac{1}{2}p(p+3) + O\left(\frac{p^3}{n}\right)\end{aligned} \tag{A.6}$$

Moreover,

$$
\begin{aligned}
\sigma_{w_n}^2 &= 2\sum_{j=1}^{2} n_j^2 \left(\frac{p}{n_j - 1} + \frac{p^2}{2(n_j-1)^2} + O\left(\frac{p^3}{n_i^3}\right)\right) - 2n^2 \left(\frac{p}{n} + \frac{p^2}{2n^2} + O\left(\frac{p^3}{n^3}\right)\right) \\
&= 2\sum_{j=1}^{2} n_j^2 \left(\frac{p}{n_j} + \frac{p}{n_j^2} + \frac{p^2}{2n_j^2} + O\left(\frac{p^3}{n_j^3}\right)\right) - 2\left(pn + \frac{p^2}{2} + O\left(\frac{p^3}{n}\right)\right) \\
&= 2np + 4p + 2p^2 - 2pn - p^2 + O\left(\frac{p^3}{n}\right) \\
&= p^2 + 4p + O\left(\frac{p^3}{n}\right)
\end{aligned} \tag{A.7}
$$

Hence, for $\lim_{n\to\infty} p_n/n = 0$, we have

$$
\begin{aligned}
\frac{f\sigma_{w_n}^2}{2\mu_{w_n}^2} &= \frac{\frac{1}{2}p(p+3)(4p+p^2+O(p^3/n))}{2\left(\frac{1}{2}p(p+3)+O(p^3/n)\right)^2} = \frac{4p^3+p^4+12p^2+7p^3+O(p^5/n)}{p^4+6p^3+9p^2+O(p^5/n)} \\
&= \frac{p^4(1+O(p/n))}{p^4(1+O(p/n))} \to 1
\end{aligned} \tag{A.8}
$$

Appendix B. Additional figure

Figure B.1 shows the graph used for the simulations described in Section 6 of the main article.

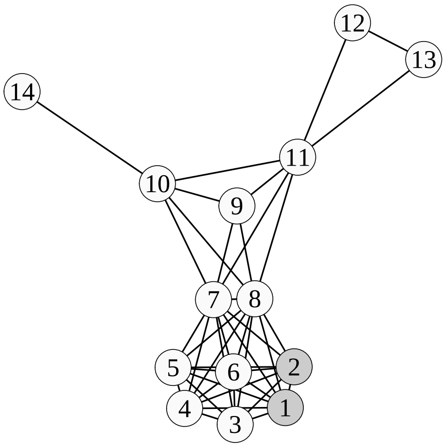

Figure B.1: Graph for the simulation study. Nodes 1 and 2 (gray) are affected by a change in the second scenario.

Appendix C. Additional simulations

Appendix C.1. Phase transition boundary

In this section, we extend the study of the phase transition boundary under the same assumptions of Figure 2, but considering different proportions of the group sample sizes. In particular, we set $n_1 = 500$ and $n_2 \in \{1000, 2500, 4000, 10000\}$ such that $n_2/n_1 \in \{2, 5, 8, 20\}$. We take $p = \lfloor n_1^{\varepsilon} \rceil$ and $\varepsilon \in \{6/24, \ldots, 23/24, 23.5/24\}$, where $\lfloor \cdot \rceil$ denotes the rounding to the nearest integer function. Figure C.2 shows the results of the empirical type-I error rate (over 1000 simulations) versus ε, for each chi-square approximation: W_n,

W_n^ρ and T_n. Simulations show that the relative size of the groups sample sizes does not influence the accuracy of the approximation.

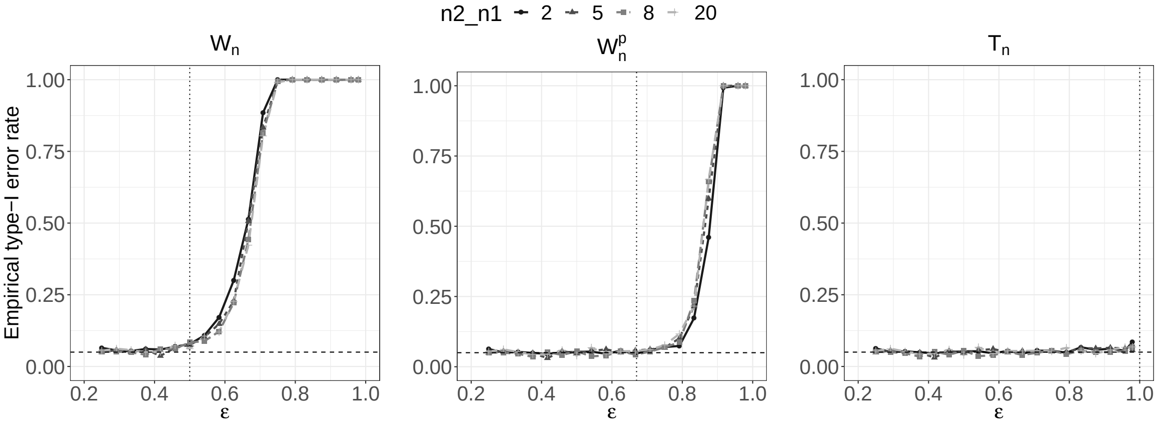

Figure C.2: Chi-square approximation of W_n, W_n^ρ and T_n. Empirical type-I error rate over 1000 simulations for $n_1 = 500$ and n_2 such that $n_2/n_1 \in \{2, 5, 8, 20\}$. Phase transition boundaries (vertical dashed lines) for the three statistics respectively: 1/2, 2/3 and 1.

Appendix C.2. Graphical setting

In this section we extend the simulation study of Section 6, showing the results under the global null hypothesis. Data were generated following the same scheme used for Table 1, but without considering any changes in the node distribution for the second condition. Results of the empirical type I error rate are shown in Table C.1. The nominal Type I error rate was set to be $\alpha = 0.05$. For the clique C_1, the empirical Type I error rate of W_n is higher than the nominal one, especially for low sample sizes. This confirms the lack of Type I error control of the W_n statistic, while T_n controls the Type I error at all sample sizes.

Appendix D. Real data application

In this Section we present a real data application. We considered the well known dataset dealing with the ABL/BCR chimera in acute lymphocytic leukemia (ALL) patients

	W_n				T_n			
n_j	10	50	100	250	10	50	100	250
C_1	0.974	0.125	0.085	0.064	0.050	0.049	0.051	0.053
$C_2\|S_2$	0.446	0.085	0.064	0.055	0.047	0.051	0.050	0.049
$C_3\|S_3$	0.169	0.059	0.058	0.050	0.048	0.044	0.050	0.048
$C_4\|S_4$	0.109	0.059	0.050	0.056	0.049	0.050	0.046	0.054

Table C.1: Type I error computed for each term of the decomposition. Number of rejected tests out of 10 thousand simulations, for different sample sizes, with significance level $\alpha = 0.05$.

(Chiaretti et al., 2005), available from the R package ALL (Li, 2021). Expression values were normalized according to rma and quantile normalization. Genes were annotated using Affymetrix Human Genome U95 Set data and duplicated Entrez IDs were averaged for each sample. Two groups of ALL patients with and without ABL/BCR genomic rearrangement (37 and 42 patients, respectively), were compared. We considered the chronic myeloid leukemia pathway, shown in Figure D.3, whose functioning is highly impacted by BCR and ABL genes.

The corresponding graph was obtained using the R package graphite (Sales et al., 2012). We finally moralized and triangulated the graph in order to obtain a decomposable graph. The obtained graph consisted of three unconnected sub-graphs, and for illustration reasons, we restricted the analysis to the largest connected component, which also included the two genes of interest, shown in Figure D.4. The final graph consists of 60 nodes and 30 cliques. Note that it is not possible to perform a global test for the equality of distributions in the two groups because the dimension of the problem is larger than the sample size. However, we exploited the decomposability of the graph and ran a test for each component. For the analysis we considered one of the 30 possible decompositions of the global null hypothesis.

Results are shown in Table D.2. Note that the hypothesis of equality of distribution is rejected only for the clique C_1. Hence, we can conclude that the two graphs are different. Moreover, the clique C_1 consists of three genes, two of which are ABL and BCR, highlighting that our method is able to highlight biologically meaningful differences between two

sets of patients.

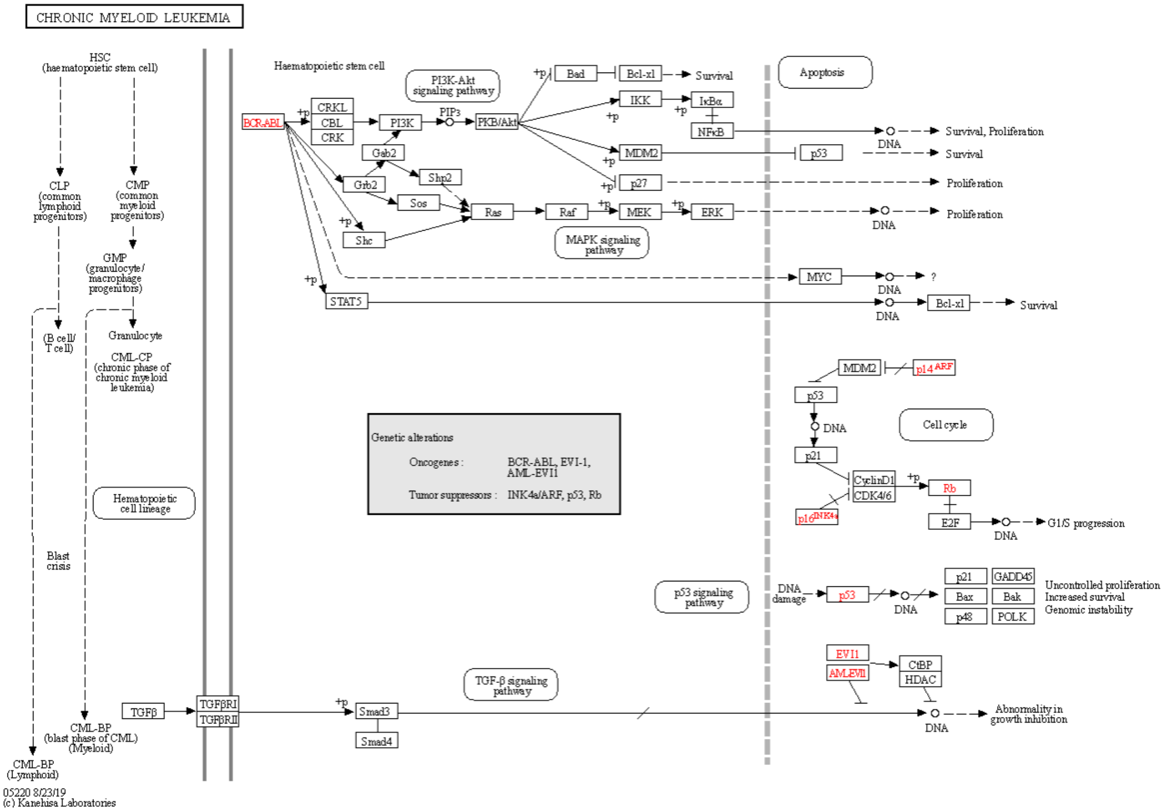

Figure D.3: Chronic myeloid leukemia pathway from KEGG (Kanehisa and Goto, 2000).

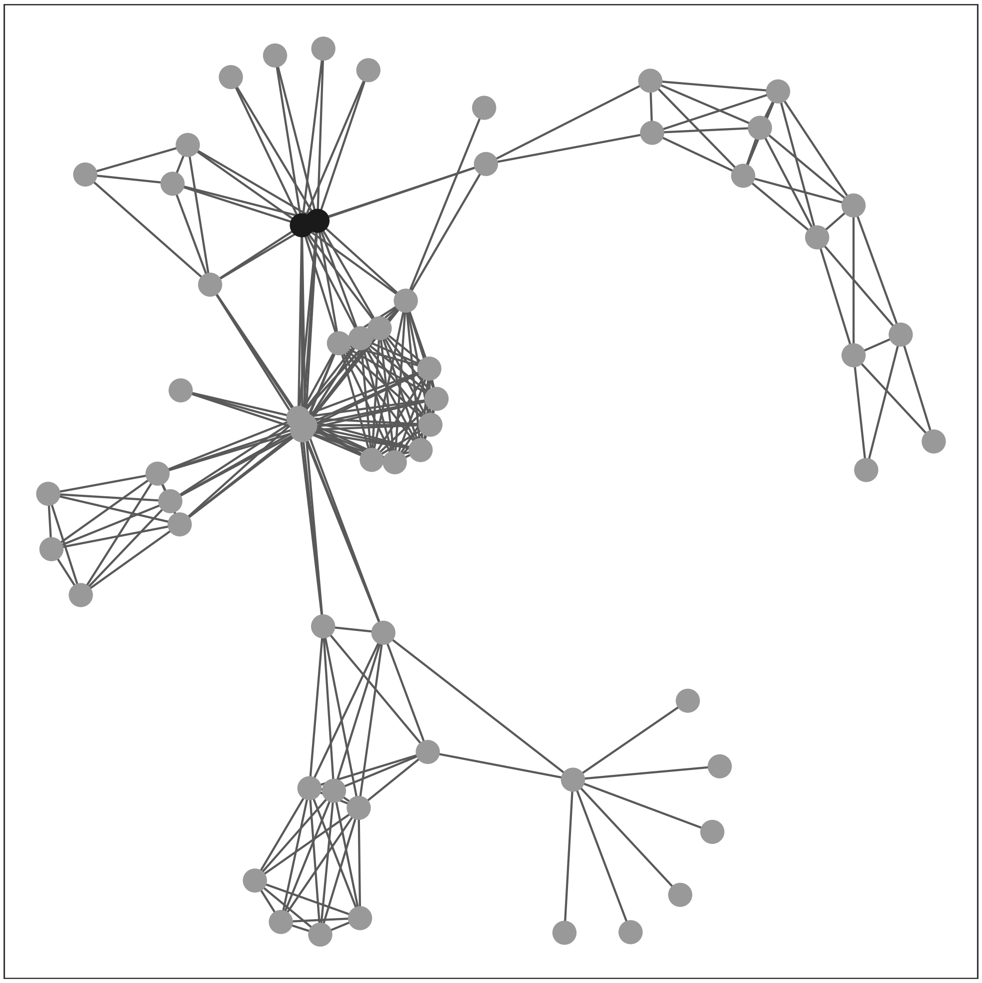

Figure D.4: Undirected graph representing the chronic myeloid leukemia pathway. Nodes in black represent the ABL and BCR genes.

	T_n	df	pvalue	adj.pvalue
C1	78.52	9	<0.001	<0.001
C2|S2	49.99	49	0.729	0.985
C3|S3	92.61	69	0.398	0.985
C4|S4	8.33	7	0.408	0.985
C5|S5	22.13	18	0.362	0.985
C6|S6	26.15	11	0.016	0.429
C7|S7	1.85	5	0.892	0.985
C8|S8	42.71	18	0.004	0.125
C9|S9	21.02	11	0.066	0.985
C10|S10	1.47	5	0.932	0.985
C11|S11	3.87	5	0.624	0.985
C12|S12	18.23	22	0.806	0.985
C13|S13	39.44	26	0.128	0.985
C14|S14	4.65	4	0.368	0.985
C15|S15	5.2	4	0.309	0.985
C16|S16	12.46	4	0.022	0.585
C17|S17	0.99	4	0.923	0.985
C18|S18	11.12	7	0.170	0.985
C19|S19	16.68	15	0.451	0.985
C20|S20	19.21	11	0.104	0.985
C21|S21	12.49	9	0.251	0.985
C22|S22	4.09	4	0.438	0.985
C23|S23	3.73	4	0.488	0.985
C24|S24	0.16	3	0.985	0.985
C25|S25	2.33	3	0.534	0.985
C26|S26	5.42	3	0.165	0.985
C27|S27	0.42	3	0.942	0.985
C28|S28	6.57	3	0.104	0.985
C29|S29	1.31	3	0.746	0.985
C30|S30	3.96	3	0.294	0.985

Table D.2: Results of the local tests on cliques. Values of the statistic T_n are reported along with the corresponding degrees of freedom (df), the raw p-values and the adjusted p-values. Adjusted p-values were obtained using the *hommel* procedure (see e.g. Goeman and Solari (2014)) in order to control the family-wise error rate.

References

Chiaretti, S., Li, X., Gentleman, R., Vitale, A., Wang, K.S., Mandelli, F., Foa, R., Ritz, J., 2005. Gene expression profiles of b-lineage adult acute lymphocytic leukemia reveal genetic patterns that identify lineage derivation and distinct mechanisms of transformation. Clinical cancer research 11, 7209–7219.

Goeman, J.J., Solari, A., 2014. Multiple hypothesis testing in genomics. Statistics in Medicine 33, 1946–1978.

Jiang, T., Qi, Y., 2015. Likelihood ratio tests for high-dimensional normal distributions. Scand. Stat. Theory Appl. 42, 988–1009.

Kanehisa, M., Goto, S., 2000. Kegg: kyoto encyclopedia of genes and genomes. Nucleic acids research 28, 27–30.

Li, X., 2021. ALL: A data package. R package version 1.34.0.

Sales, G., Calura, E., Cavalieri, D., Romualdi, C., 2012. graphite-a bioconductor package to convert pathway topology to gene network. BMC bioinformatics 13, 1–12.
